# Cross-linking cellulose nanofibrils for potential elastic cryo-structured gels

**DOI:** 10.1186/1556-276X-6-626

**Published:** 2011-12-12

**Authors:** Kristin Syverud, Harald Kirsebom, Solmaz Hajizadeh, Gary Chinga-Carrasco

**Affiliations:** 1 Paper and Fibre Research Institute (PFI), Høgskolerringen 6b, Trondheim, NO-7491, Norway; 2 Department of Biotechnology, Lund University, P.O. Box 124, Lund, SE-22100, Sweden

**Keywords:** cellulose nanofibrils, MFC, cryogelation, cross-linking

## Abstract

Cellulose nanofibrils were produced from *P. radiata *kraft pulp fibers. The nanofibrillation was facilitated by applying 2,2,6,6-tetramethylpiperidinyl-1-oxyl-mediated oxidation as pretreatment. The oxidized nanofibrils were cross-linked with polyethyleneimine and poly *N*-isopropylacrylamide-*co*-allylamine-*co*-methylenebisacrylamide particles and were frozen to form cryo-structured gels. Samples of the gels were critical-point dried, and the corresponding structures were assessed with scanning electron microscopy. It appears that the aldehyde groups in the oxidized nanofibrils are suitable reaction sites for cross-linking. The cryo-structured materials were spongy, elastic, and thus capable of regaining their shape after a given pressure was released, indicating a successful cross-linking. These novel types of gels are considered potential candidates in biomedical and biotechnological applications.

## Background

### Cellulose nanofibrils

The main raw material for the production of microfibrillated cellulose [MFC] is cellulose fibers, produced from wood by chemical pulping. Properly produced MFC contains a major fraction of cellulose nanofibrils [1]. Nanofibrils are composed of bundles of cellulose molecules, arranged in crystalline and amorphous areas. Nanofibrils have threadlike shapes, with diameters in the nanometer scale (< 100 nm), with high aspect ratio and high specific surface area. The fibrillated material retains many of the advantageous properties of cellulose fibers, such as high strength and the ability to self-assemble by making strong inter-fibril bonds. The small dimensions and the large specific surface area open up for applications that may not yet be foreseen. Several recent publications demonstrate how the strength properties of cellulose nanofibrils can be utilized for various purposes, e.g., in nanocomposites [1-6], to improve strength properties of paper [7,8], in thin films with high strength [9] and with added functionality such as antimicrobial activity [10].

Nanofibrils have hydroxyl groups on their surfaces, which can be used as targets for surface modification. Pretreatment of cellulose fibers with 2,2,6,6-tetramethylpiperidinyl-1-oxyl [TEMPO] prior to fibrillation introduces carboxylic acid groups and small amounts of aldehyde groups (0.2 to 0.3 mmol/g) [11], which can react easily with amines [12].

### Cryogelation

Subjecting a solution or suspension to temperatures below the freezing point but above the eutectic point of the system leads to the formation of a two-phase system, with one solid and one liquid phase. When ice crystals form, any solutes or particles are expelled into a non-frozen phase, which forms around the crystals. In cryogelation, the gelation process occurs in the non-frozen phase, and hence, a material is formed under apparently frozen conditions [13]. The gelation can either occur through chemical cross-linking, polymerization reactions, or through non-covalent interactions. However, it is crucial that the interactions do not reverse when the sample thaws since that would make it impossible to form a material through cryogelation. Thawing the sample results in melting of the ice crystals while the material, formed through gelation, retains its shape. A macroporous material can thus be formed, in which the pores are a replica of the ice crystals [13].

Pores in materials formed through cryogelation are interconnected and normally exhibit diameters between 1 and 100 μm, depending on freezing temperatures and composition of the starting mixture. Cryogelation does not require a freeze-drying step in order to produce a macroporous structure. The technique is only based on a freeze-thawing process. Cryogels are highly macroporous and often elastic materials, which can make them suitable in applications where traditional hydrogels would not be applicable. These gels have been used for biotechnological applications such as chromatography materials to process particle-containing fluids or enzyme immobilization [14]. Within biomedical applications, cryogels are being used in scaffolds for the cultivation of mammalian cells in tissue engineering applications [15].

The application of cellulose nanofibrils as a main component, in combination with polymers/particles as cross-linkers to form macroporous hydrogels, has not been investigated yet. It is expected that such gels can have a major potential within, e.g., biomedical applications. This study thus focuses on the ability of cellulose nanofibrils combined with cryogelation to produce cryo-structured gels with elastic properties. Two different routes will be applied for cross-linking, i.e., reactions with polyethyleneimine [PEI] and poly *N*-isopropylacrylamide-*co*-allylamine-*co*-methylenebisacrylamide [pNIPA] particles.

## Methods

### Production of cellulose nanofibrils

Two series of nanofibril qualities were produced from 100% *P. radiata *kraft pulp fibers. One of the series was chemically pretreated by using TEMPO-mediated oxidation, according to Saito et al. [11]. The other series was homogenized without pretreatment. The fibers were homogenized with a Rannie 15 type 12.56X homogenizer operated at 1,000 bar pressure. The pulp consistency during homogenizing was 0.5%. Samples of the fibrillated materials were collected after five passes through the homogenizer. For details, see the work of Syverud et al. [16].

### Cross-linking nanofibrils

The nanofibrillated material had a concentration of approximately 0.5% (*w*/*v*). PEI (0.4% *w*/*v*; molecular weights 600 and 1,800 g/mol) from PolyScience (Niles, IL, USA) was added to this suspension. This mixture was thereafter frozen at -12°C and stored for 16 h; after which, the samples were thawed at room temperature, and the obtained gels were washed with water.

The second route for preparing gels consisted the adding of pNIPA particles (0.04% *w*/*v*) [17] to the nanofibril suspension. Allylamine and *N,N'*-methylene-bisacrylamide were purchased from Sigma-Aldrich (Steinheim, Germany), and *N*-isopropylacrylamide was from Acros (Geel, Belgium). The mixture was thereafter frozen at -12°C and stored for 16 h. The samples were then thawed at room temperature, and the obtained gels were washed with water.

### Characterization

The prepared samples were cut into a 2-mm-thin disc and fixed in 2.5% *w*/*v *glutaraldehyde in 0.1 M sodium phosphate buffer (pH 7.4) overnight at +4°C. The samples were thereafter stepwise dehydrated in ethanol (0%, 25%, 50%, 75%, 96%, 99.6%) and then critical-point dried. The dried samples were sputter-coated with gold/palladium (40/60) and examined using a JEOL JSM-5000LV scanning electron microscope [SEM] (JEOL Ltd., Akishima, Tokyo, Japan). In addition, the cross-linked nanostructures were freeze-dried and assessed with a Zeiss Ultra field-emission scanning electron microscope [FESEM] (Carl Zeiss AG, Oberkochen, Germany) at various magnifications.

The mechanical stability of the cryo-structured gels was assessed using a texture analyzer (XT2i, Stable Micro Systems, Godalming, England), using a 5-kg load cell and a cylindrical probe (25 mm in diameter).

## Results and discussion

**Figure 1:**
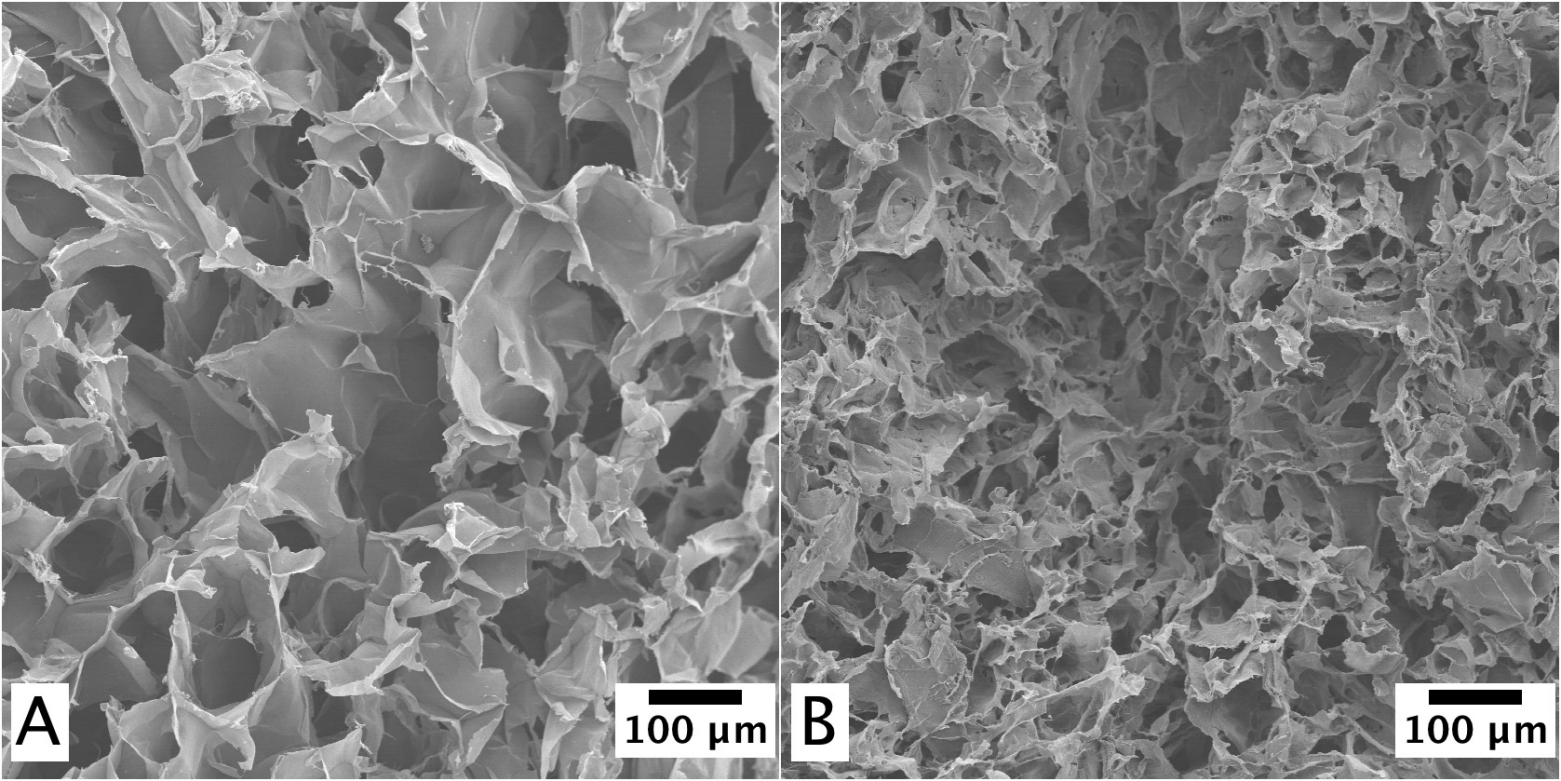
**SEM images of gel prepared from nanofibrils**. The nanofibrils have been linked with (**A**) 1,800 g/mol PEI and (**B**) pNIPA particles.

Non-oxidized nanofibrils did not form into gels when the nanofibrils were mixed with PEI. The lack of aldehyde groups on these fibrils does not allow any reaction between the fibrils and the PEI; therefore, the obtained results were not unexpected. However, the addition of PEI to the oxidized nanofibrils resulted in the formation of gels (Figure 1). It is likely that the aldehyde groups enabled the reaction with the added PEI, which formed stable inter-fibril bonds. It is worth to mention that from the physical observation of the gels, the addition of 1,800 g/mol PEI produced more stable and spongy gels than the addition of 600 g/mol PEI under compression. PEI acts as a cross-linker between the fibrils, and thus, the length of the cross-linker will influence the properties of the formed material.

**Figure 2:**
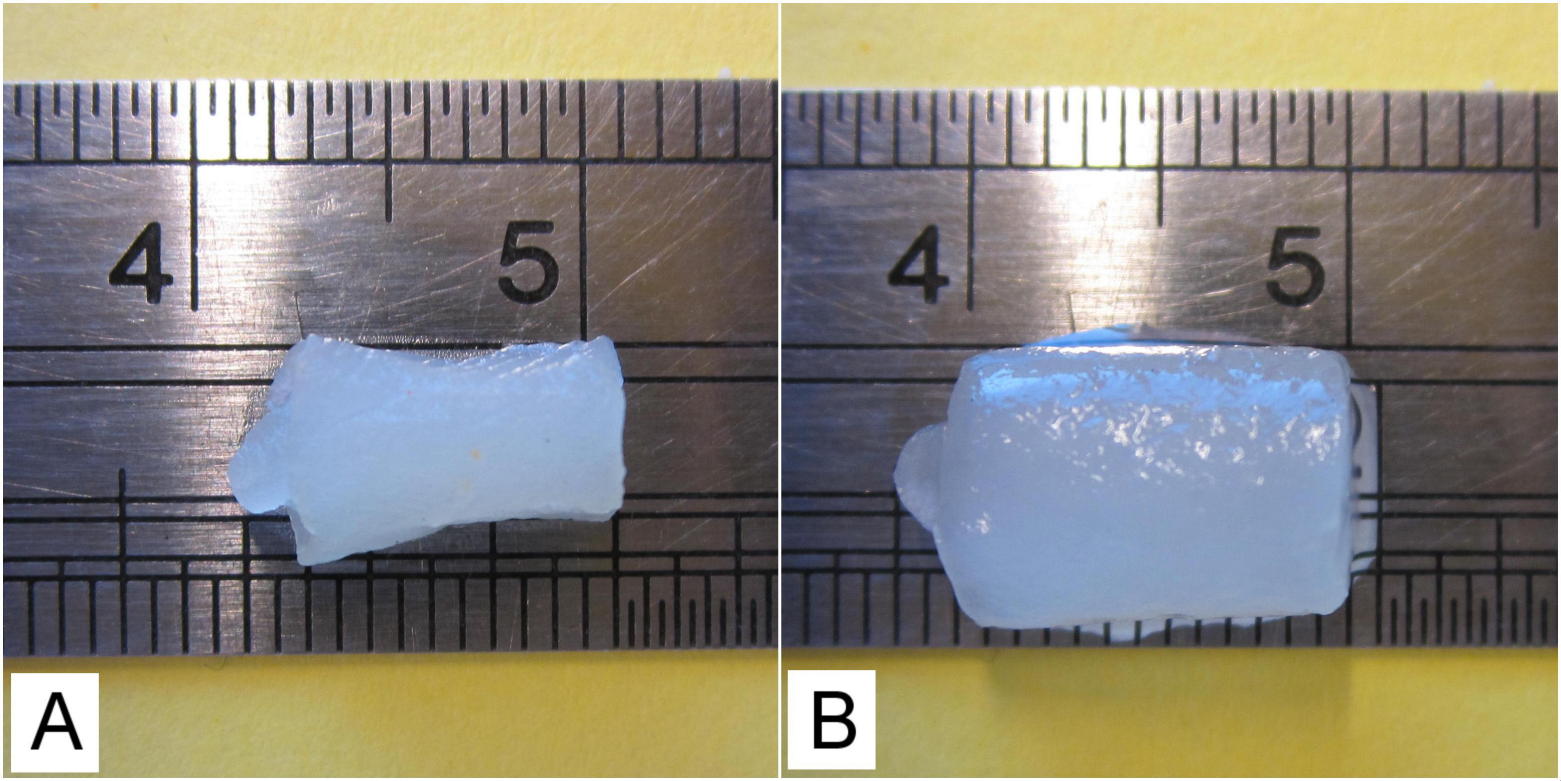
**Digital photos of nanofibril pNIPA cryo-structured gel**. (**A**) Photo of gel at room temperature. (**B**) Photo of gel at 60°C.

The addition of pNIPA particles (size approximately 125 nm) to the oxidized nanofibril suspension also resulted in the formation of stable and spongy gels (Figure 1B). The amino groups on the pNIPA particles, due to the allylamine, made it possible for the particles to react with the nanofibrils. Using pNIPA particles as a cross-linker can introduce temperature-responsive properties of the material [18]. It is well known that pNIPA collapses at temperatures above the lower critical solution temperature [LCST], which is about 32°C [19]. Therefore, at temperatures above the LCST, the cryo-structured material with cellulose nanofibrils will undergo a volumetric shrinking (Figure 2). The cryo-structured nanofibril gel shrank in all three dimensions due to the presence of the pNIPA particles in the gel.

**Figure 3:**
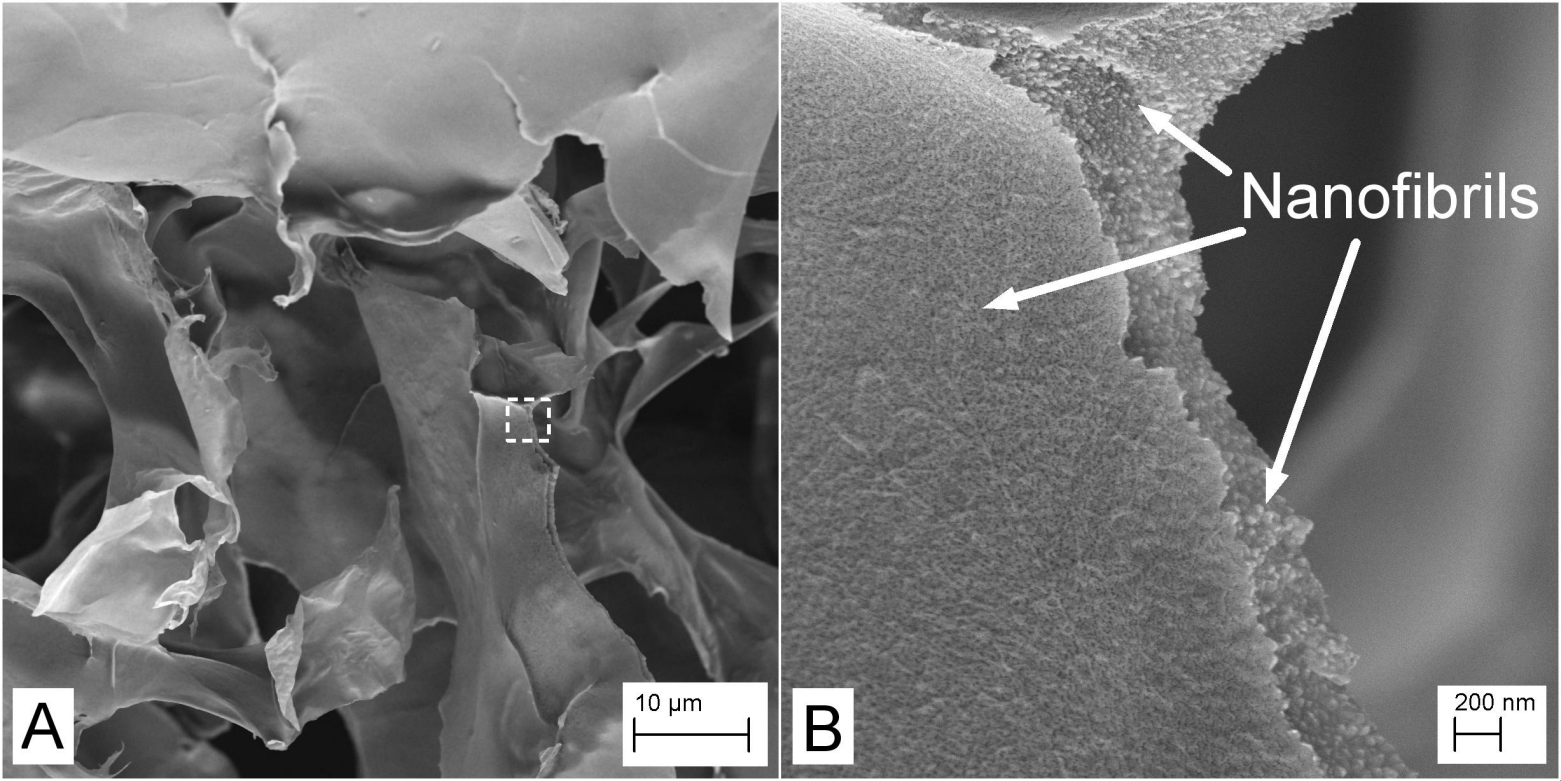
**FESEM images of a gel prepared from nanofibrils mixed with pNIPA particles**. Images acquired at relatively (**A**) low and (**B**) high magnifications. The image in (B) has been acquired from the area marked with a dotted rectangle in the image in (A). Note the nano-sized fibrils forming the network structure.

**Figure 4:**
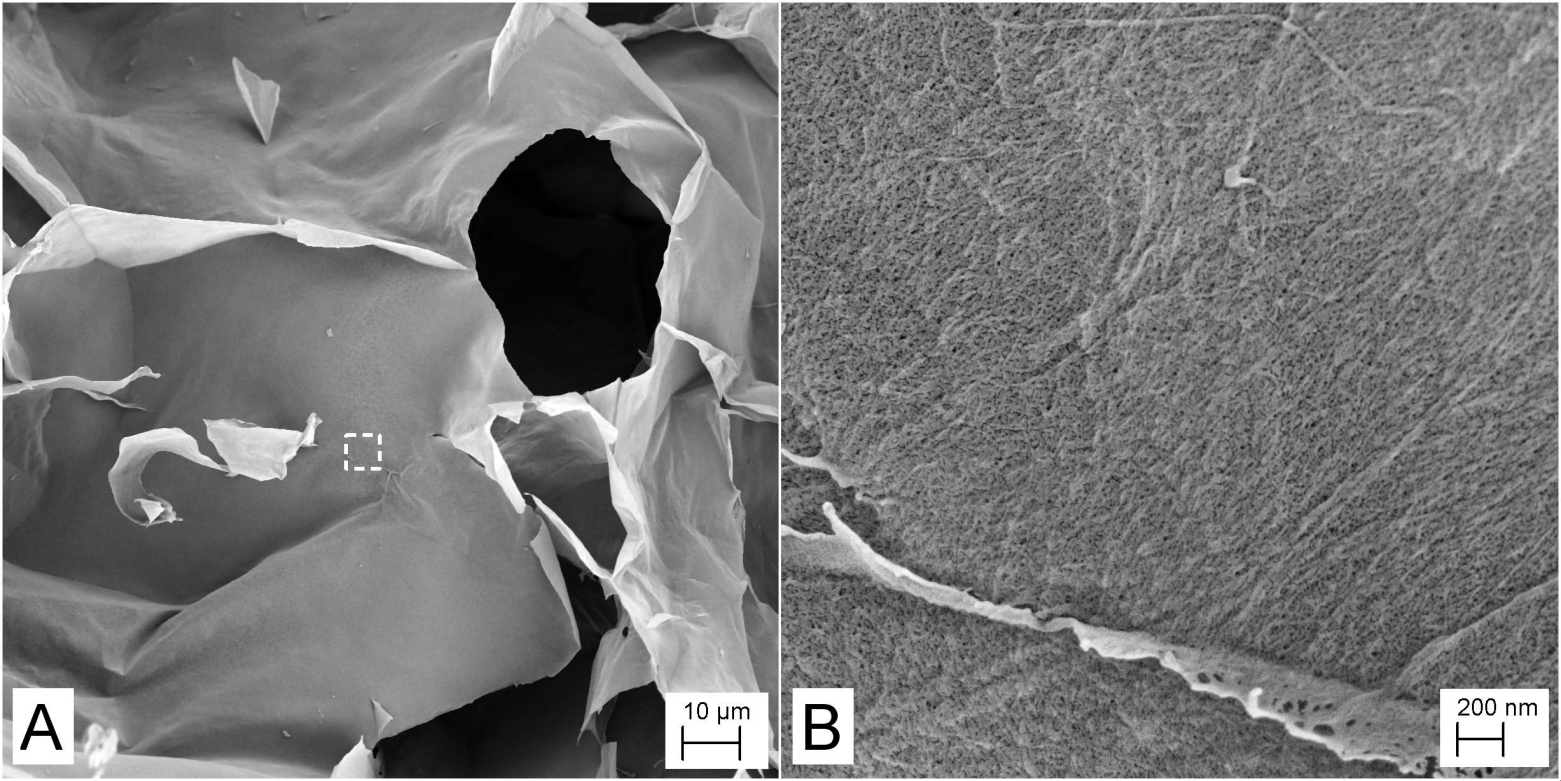
**FESEM image of a gel prepared from nanofibrils linked with 1,800 g/mol PEI**. Images acquired at relatively (**A**) low and (**B**) high magnifications. The image in (B) has been acquired from the area marked with a dotted rectangle in the image in (A). Note the nano-sized fibrils forming the network structure.

High-resolution FESEM images were acquired to reveal the nanofibril structure and assembly in the gels (Figures 3 and 4). Note the relatively thin layers revealed in Figures 3B and 4B. The layers are composed of nanofibrils with diameters < 20 nm, as has been reported recently for this fibrillated material [20]. Such nanofibrils are clearly exposed in a fracture area visualized at high-resolution (Figure 3B).

**Figure 5:**
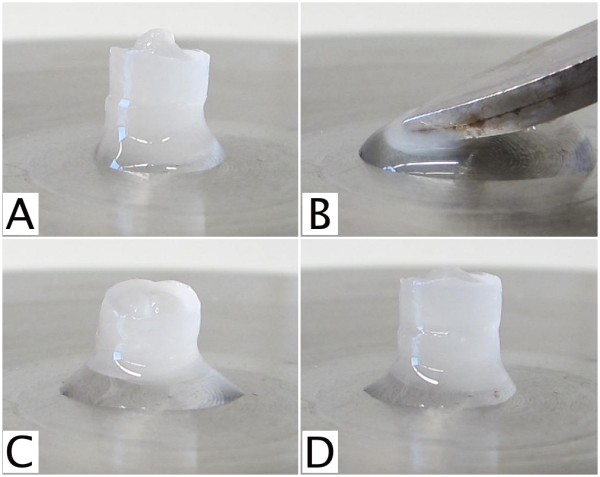
**Digital photographs of a gel prepared from cellulose nanofibrils cross-linked with 1,800 g/mol PEI**. (**A**) Before compression, (**B**) during compression, and (**C**) after pressure have been released. (**D**) The gel regains its original shape.

**Figure 6:**
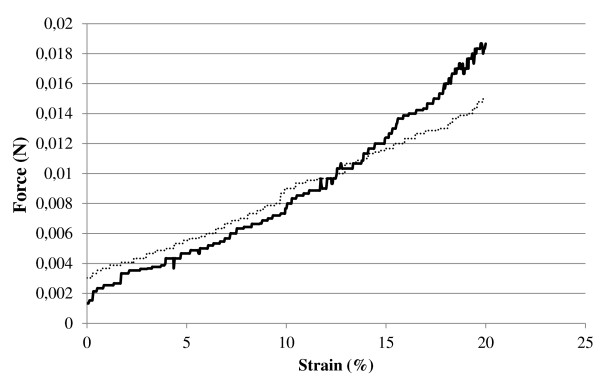
**Force plotted against strain**. Cellulose nanofibrils cross-linked with PEI (dark black line) and cellulose nanofibrils with pNIPA particle cryo-structured gels (dash line).

Figure 5 shows photographs of the gels obtained after cross-linking with 1,800 g/mol PEI. It displays a sponge-like property in which the water can easily be squeezed out by pressing the cryo-structured gel. The gel easily regains its shape after the pressure is released. Similar results were obtained when the gel was cross-linked with pNIPA particles. The mechanical stability of the cryo-structured gels were determined by a texture analyzer, and from force-distance curve, mechanical elasticity of the gels can be derived. Data show that even after compression of the gels, they will be expanded to their original form (Figure 5). The gels were compressed up to 20% of their height for the mechanical testings (Figure 6).

The results presented in this study indicate that the nanofibrils are interesting building blocks to prepare cryo-structured materials. Based on the sponge-like property of these cryo-structured materials, we foresee high-tech applications, such as modified macroporous structures in biomedical and biotechnology areas.

## Conclusions

Oxidized nanofibrils, produced from *P. radiata *pulp fibers, were cross-linked with PEI and pNIPA particles in order to form cryo-structured gels. Due to a successful cross-linking, the nanofibrils formed stable 3-D networks. The cryo-structured materials were spongy, elastic, and thus capable of regaining their shape after a given pressure was released. Such characteristics propose the cryo-structured nanomaterials as most promising within biomedicine and biotechnology applications.

## Abbreviations

FESEM: field emission scanning electron microscope; LCST: lower critical solution temperature; MFC: microfibrillated cellulose; PEI: polyethyleneimine; pNIPA: poly *N*-isopropylacrylamide-*co*-allylamine-*co*-methylenebisacrylamide; SEM: scanning electron microscope.

## Competing interests

The authors declare that they have no competing interests.

## Authors' contributions

HK has been involved in planning and synthesizing the cryo-structured materials and in writing and revising the manuscript. KS has made a substantial contribution to the conception of the experiments, has been involved in the production and characterization of cellulose nanofibrils, and in revising the manuscript critically for important intellectual content. SH has been involved in the production of pNIPA particles and synthesis of cryo-structured gels from cellulose nanofibrils and particles, performed the texture analysis, and contributed in revising the manuscript. GCC has been involved in the production and characterization of cellulose nanofibrils, performed the FESEM analysis of the cryo-structured gels, drafted the manuscript, and performed the corresponding revisions. All authors have read and approved the final manuscript.
